# Reduced Anxiety-Like Behavior and Altered Hippocampal Morphology in Female p75NTR^exon IV−/−^ Mice

**DOI:** 10.3389/fnbeh.2016.00103

**Published:** 2016-06-01

**Authors:** Zoe Puschban, Anupam Sah, Isabella Grutsch, Nicolas Singewald, Georg Dechant

**Affiliations:** ^1^Department of Neuroscience, Innsbruck Medical UniversityInnsbruck, Austria; ^2^Department of Pharmacology and Toxicology, Institute of Pharmacy, Center for Molecular Biosciences (CMBI), Leopold-Franzens University of InnsbruckInnsbruck, Austria

**Keywords:** p75NTR^exon IV−/−^ mouse, anxiety, ventral hippocampus, dorsal hippocampus, sex difference

## Abstract

The presence of the p75 neurotrophin receptor (p75NTR) in adult basal forebrain cholinergic neurons, precursor cells in the subventricular cell layer and the subgranular cell layer of the hippocampus has been linked to alterations in learning as well as anxiety- and depression- related behaviors. In contrast to previous studies performed in a p75NTR^exon III−/−^ model still expressing the short isoform of the p75NTR, we focused on locomotor and anxiety–associated behavior in p75NTR^exon IV−/−^ mice lacking both p75NTR isoforms. Comparing p75NTR^exon IV−/−^ and wildtype mice for both male and female animals showed an anxiolytic-like behavior as evidenced by increased central activities in the open field paradigm and flex field activity system as well as higher numbers of open arm entries in the elevated plus maze test in female p75NTR knockout mice. Morphometrical analyses of dorsal and ventral hippocampus revealed a reduction of width of the dentate gyrus and the granular cell layer in the dorsal but not ventral hippocampus in male and female p75NTR^exon IV−/−^ mice. We conclude that germ-line deletion of p75NTR seems to differentially affect morphometry of dorsal and ventral dentate gyrus and that p75NTR may play a role in anxiety-like behavior, specifically in female mice.

## Introduction

p75 neurotrophin receptor (p75NTR) is a member of the TNF-receptor superfamily that regulates cell survival, apoptosis, neurite outgrowth and myelination (Bandtlow and Dechant, 2004). In contrast to the widespread expression of p75NTR throughout development, in the adult CNS of rodents it is restricted to the basal forebrain cholinergic neurons and to precursor cells in the subventricular cell layer as well as the subgranular layer of the dentate gyrus (Bernabeu and Longo, 2010; Martinowich et al., 2012). Two mouse models carrying a targeted mutation in the gene encoding p75NTR have been generated (Lee et al., 1992; von Schack et al., 2001). The most widely studied model developed by Lee et al. (1992) has targeted exon III of the p75NTR gene preventing the expression of the full-length receptor, however, this model still expresses the short protein isoform of p75NTR (von Schack et al., 2001) that arises from alternative splicing of exon III in the p75NTR locus. Targeting exon IV led to a complete ablation of both naturally occurring p75NTR isoforms (von Schack et al., 2001). Contradicting results have been obtained regarding effects of the p75NTR^exon III−/−^ mutation on number and size of basal forebrain cholinergic neurons, reporting either increase, decrease or no change (Yeo et al., 1997; Peterson et al., 1999; Greferath et al., 2000, 2012). In p75NTR^exon IV−/−^ mice an increase in number of cholinergic basal forebrain neurons of the medial septum was reported (Naumann et al., 2002). This increase was also seen in a p75NTR conditional knockout mouse, where p75NTR was removed from postmitotic cholinergic basal forebrain neurons from postnatal day 4 on (Boskovic et al., 2014), resulting in an increased cholinergic innervation of the cortex but not the hippocampus. In p75NTR^exon III−/−^ and p75NTR^exon IV−/−^ mice, germ line deletion of p75NTR caused a strong increase of the cholinergic fiber density as well as increased densities of dendritic spines in the dentate gyrus of adult mice (Dokter et al., 2015; Poser et al., 2015). Morphometric analyses of hippocampal formation and associated tests for anxiety and depression as well as locomotor behavior in p75NTR^exon III−/−^ mice have led to conflicting results. Different background strains of the animals used as well as differences in age and sex of the animals tested and experimental settings might account for these discrepancies (Dokter et al., 2015). Catts et al. (2008) observed a significant reduction in the dentate gyrus volume and width of the granular cell layer in male p75NTR^exon III−/−^ mice, (Catts et al., 2008; Colditz et al., 2010). They did not observe any obvious anatomical or physiological defects in the p75NTR^exon III−/−^ mice, except for bulbous toes on the hind paws, which did not interfere with general locomotor activities. In behavioral tests the male p75NTR^exon III−/−^ mice showed impairments in the novelty-suppressed feeding test for assessment of depressive-like behavior, whereas there was no difference in the forced swim test and an even longer mobility time in the tail suspension test (Catts et al., 2008). In this study, no significant effect of the mutation was observed in tests for anxiety-like behavior, including elevated-plus maze test and light dark test. This is in contrast to Martinowich et al. (2012), who saw no difference between male wildtype and knockout animals in tests of baseline depressive-like behavior (forced swim test, tail suspension test) but a significant influence of the mutation on baseline anxiety-like behavior. The laboratory of von Bohlen und Halbach (Dokter et al., 2015; Poser et al., 2015) reported an increase of the granular layer width of dentate in p75NTR^exon III−/−^ and an increase of both granular and molecular layer width in p75NTR^exon IV−/−^ animals. Behavioral analyses were restricted to male p75NTR^exon III−/−^, where they reported increased locomotion and velocity but no difference in number of center entries in the open field paradigm.

The aim of the present study, was to characterize motor and anxiety-related behavior of p75NTR^exon IV−/−^ mice including animals of both sexes. Behavioral analyses in p75NTR^exon IV−/−^ have so far been restricted to observation of obvious gate deficits, described as “mud-walking” gait and stretching of their posterior extremities pointing upward when held by their tails (von Schack et al., 2001). In our hands ablation of the p75 NTR resulted in significantly reduced levels of anxiety-like behavior selectively in female p75NTR^exon IV−/−^ mice. Morphometric analysis of the hippocampus was performed separately for dorsal and ventral hippocampus, taking into account that regional differences regarding hippocampus-dependent functions exist, with anxiety being mainly associated with the ventral hippocampus (Bannerman et al., 2003a,b).

## Materials and Methods

### Animals

Adult male and female p75NTR^exon IV−/−^ mice and C57 BL6 wildtype control mice (Harlan Winkelmann, Germany) were used in this study. Ethical permit was obtained by the Austrian Ministery of Science (BMWF-66.011/0090-II/10b/2009). Animals were housed in the Central Animal Facility of the Medical University of Innsbruck (Zentrale Versuchstieranlage, Innsbruck, Austria) in a temperature controlled room, maintained on a 12-h light/dark cycle and allowed free access to food and water. Efforts were made to minimize the number of animals used while maintaining statistically valid group numbers and to minimize pain and stress to the animals. When female mice were tested cycling stages were defined.

### Behavioral Tests

All the behavioral testings were performed on separate days in male and female mice.

#### Home Cage Locomotor Activity

Three month old mice of both sexes and genotypes (*n* = 8 for male and female wildtype—WT and male knockout—P75KO, *n* = 6 for female P75KO) were used. One animal was omitted from the analysis. Activity, i.e., locomotion and exploration, significantly varies depending on the scene of measurement. Activity in the home cage is predominantly influenced by activity rhythm, in contrast to behavioral tests, where mice are placed in an unfamiliar environment, which affects fear and anxiety due to neophobia. Mice were single housed 12 h before activity monitoring started. Measurement started at the beginning of the dark cycle after 12 h of habituation.We quantified home cage activity via an automated system (Inframot; TSE, Bad Homburg, Germany) over a period of two light and three dark cycles as previously described (Singewald et al., 2004; Borrie et al., 2014; Sotnikov et al., 2014). Measurement was started at the beginning of the dark cycle after 12 h of habituation. Eight animals were tracked simultaneously, each in a type 3 *Makrolon* cage (265 × 150 × 420 mm). The system monitored the activity of the mice by sensing the body heat image, i.e., infra-red radiation, and its spatial displacement over time. No movements were monitored when the mice were inactive, sleeping or during moderate self-grooming. Data were collected in bins of 1 min and were subsequently pooled to 1-h intervals.

#### Open Field Test

The same set of animals underwent open field testing. The open field consists of a plastic box (41 × 41 × 41 cm) equipped with an automatic activity monitoring system (TruScan, Coulbourn Instruments, MA, USA). The area of the open field, illuminated with 150 lux, was divided into a 28 × 28 cm central zone and a surrounding border zone. Mice were placed individually into the periphery of the open field to allow exploration for 10 min. The following anxiety-related parameters were recorded: center distance traveled, center entries and center time in seconds, rearing number and time (Sartori et al., 2012), and total distance and total moving time were used to assess general locomotion.

#### Elevated Plus Maze Test

Two days after the open field testing, the elevated plus maze test was performed as described previously (Busquet et al., 2010) on the same set of male and female mice of both genotypes. The apparatus was elevated 73 cm above the floor and exposed to red light (15 lux) while the room was dimly lit with yellow light (50 lux). At the beginning of each trial, a mouse was placed onto the central area of the maze, facing a closed arm. Mice were tested for a period of 5 min and their movements on the maze were tracked and subsequently analyzed by the TSE VideoMot2 system (TSE, Technical and Scientific Equipment GmbH, Germany). Following parameters were assessed: open and closed arm entries, open and closed arm time, latency to enter open arm for the first time, total arm entries and % of time spent in open arm.

#### Flex Field Test

In a separate cohort of 16 female WT and 16 female P75KO mice at the age of 7–8 months, flex field test was conducted in a 42 × 42 × 32 cm photobeam-equipped enclosure (Flexfield Activity System, San Diego Instruments, CA, USA), made of white floor and clear plastic walls. Briefly, the enclosure is crossed in a grid pattern by 48 photobeams, which allows monitoring and real-time counting of horizontal and vertical locomotor activity. The test was performed at 7 p.m. over a time period of 15 min in a dark room that was completely isolated from external noises and light during the test period analog to Scherfler et al. (2005) with minor modifications. Total distance traveled, horizontal, rearing and central vs. peripheral activities were measured.

### Histological Analysis

#### Tissue Processing

Two days after the elevated plus maze testing, animals were perfused under deep thiopental anesthesia with phosphate-buffered saline at room temperature for 2–3 min followed by ice-cold 4% paraformaldehyde in phosphate-buffered saline with a pH of 7.4. Brains were quickly removed and immersed in 25% sucrose until they sank. Brains were then frozen in isopentane on dry ice and stored at −80°C. For hippocampal analysis sections were cut on a cryostat first in a horizontal plane (for assessment of the ventral hippocampus) from a level of 0.92 mm in relation to the interaural line to a level of 2.20 mm according to mouse brain atlas (Franklin and Paxinos, 2007) in repetitive series: 5 × 40 μm collected free-floating and 5 × 8 μm mounted onto gelatine-coated slides. For analyses of the dorsal hippocampus the remaining brain was mounted to the cryotome in a way that allows coronar sections. The section series remained the same as for the horizontal slices starting with collection of the sections at an anterior-posterior (AP) level in relation to bregma −0.94 mm until an AP level of −3.80 mm, thereby spanning the entire dorsal hippocampus.

#### Morphometry of Dorsal and Ventral Hippocampus

One series of 40 μm both horizontal and coronar sections was stained with cresyl violet. Sections were further processed for morphometric analyses of the hippocampus. Surface measurements were performed on six sections for both dorsal and ventral hippocampus of male and female WT and p75KO mice, respectively. To this end, a computer assisted image analysis system (Axio Vision AC, Rel 4.1) was employed. Using a 2.5× objective the areas of interest were outlined with a mouse-controlled cursor. Morphometric analysis was performed on both hemispheres strictly according to the mouse brain atlas (Franklin and Paxinos, 2007, Supplementary Figure 1). Following parameters were assessed for the dorsal part of the hippocampus: area of the dentate gyrus, area of the hippocampal formation without the dentate gyrus, area of the granular cell layer, width of the granular and molecular cell layer of the dentate gyrus, width of CA1 and CA3, width of the dentate gyrus (b) and width of the hippocampus proper (a) in order to calculate the ratio of a to b. Parameters measured in the ventral hippocampus included: area of the dentate gyrus, area of the hippocampal formation without the dentate gyrus, area of the granular cell layer, width of the granular and molecular cell layer of the dentate gyrus, width of CA1 and CA3, width of the dentate gyrus (b) and width of the hippocampus excluding the dentate gyrus (a). Cross sectional area of the striatum was measured to control for shrinkage artifacts. The experimenter was blinded as to genotype and gender of the animals.

#### DAPI Cell Counting

Standard DAPI staining was performed on complete series of 8 μm sections. Cells counts in the granular cell layer, CA1 and CA3 area of the ventral hippocampus of DAPI stained sections were performed using a 40× objective and by the means of a grid (one square corresponds to 25 μm × 25 μm). Cells were counted over a length of 125 μm and the complete width of the corresponding areas.

### Statistics

Statistica Software, Statsoft Inc., OK, USA was used. The behavioral parameters and cell counts were statistically analyzed by using an unpaired *t*-test. In case the data set failed the normality or equal variance test, Mann-Whitney rank sum test was used instead. Home cage analysis was conducted using a repeated-measures analysis of variance (ANOVA) with genotype as an independent and time as a dependent factor. A Duncan’s *post hoc* comparisons test was applied following the repeated-measures ANOVA. Statistical significance was set at *p* < 0.05. For correlation analysis Pearson product-moment correlation was applied. For the figures, the individual value from each animal in the WT group was divided by the group mean which was expressed as percentage (%). This analysis resulted in the % value for each animal, using which group Mean + SEM for WT was calculated (Santarelli et al., 2003).

## Results

### Behavior

#### Home Cage Locomotor Activity

**Figure 1:**
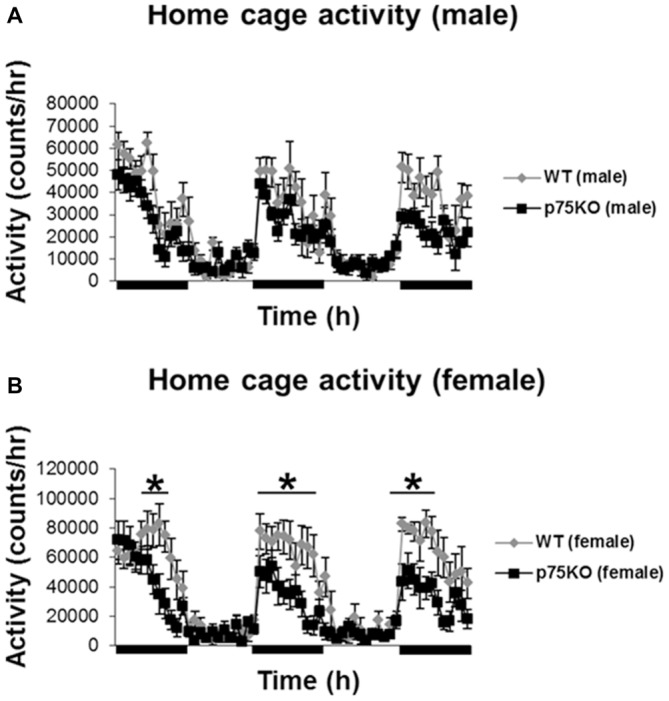
**Altered activity during active nocturnal phase in the home cage test in female p75NTR^exon IV−/−^ mice (B).** Black bars indicate nocturnal/dark periods. Results of male mice are depicted in **(A).** wildtype (WT), gray bar; p75 neurotrophin receptor knockout (p75KO), black bar. Data are presented as Mean ± SEM, *p < 0.05.

Homecage activity was used to evaluate the baseline activity of the WT and p75KO mice and the analysis was conducted using repeated measures ANOVA. As expected, there was a significant effect of circadian phases on spontaneous locomotor activity in both experimental groups (*p* < 0.001) with a pronounced elevation in the dark phase (male: *F*_(59,826)_ = 15.4, *p* < 0.001) female: *F*_(59,649)_ = 16.8, *p* < 0.001). Futhermore, a significant genotype × time effect: *F*_(59,649)_ = 2.57, *p* < 0.001 in the female p75KO (compared to WT), but not male, mice was observed. Female p75KO (compared to WT) also showed reduced activity (*F*_(1,11)_ = 6.6, *p* < 0.05) during the dark phase (active period), but not during the light phase (inactive period). During the light phase spontaneous locomotor activity was generally low in both groups (Figures 1A,B).

#### Open Field

**Figure 2:**
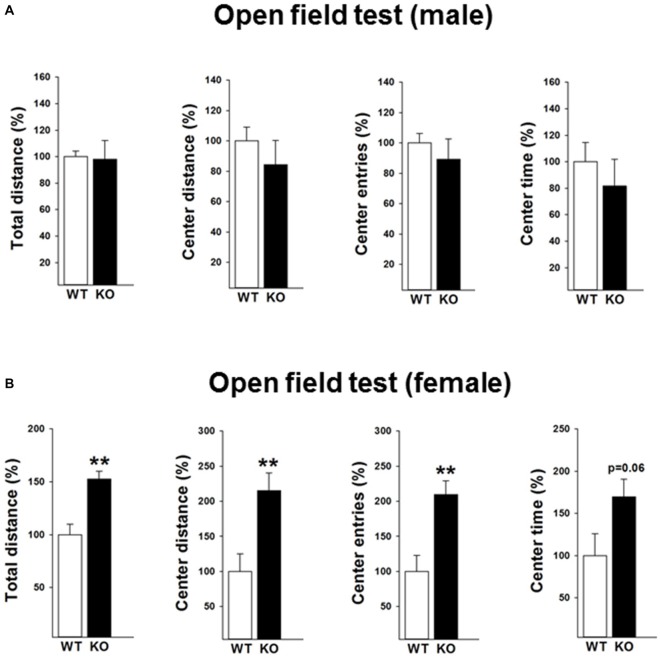
**Reduced anxiety in the open field test in the female p75NTR^exon IV−/−^ mice (B).** They show significantly higher total distance and center distance traveled and more center entries. An increased center time (trend) was also observed. Results of male mice are depicted in **(A)**. WT, white bar; KO, black bar. WT data are normalized to 100%. Group Mean + SEM; Male WT: total distance = 2177.8 + 92.4 cm, center distance = 353.4 + 31.8 cm, center entries = 30.3 + 1.9, center time = 114.1 + 16.6 s, Male p75KO: total distance = 2136.8 + 307.34 cm, center distance = 298.5 + 55.7 cm, center entries = 27.1 + 4.0, center time = 93.2 + 23.1 s, Female WT: total distance = 1760.2 + 171.9 cm, center distance = 143.6 + 36.1 cm, center entries = 14.6 + 3.3, center time = 50.1 + 12.9 s, Female p75KO: total distance = 2687.9 + 128.0 cm, center distance = 309.1 + 36.3 cm, center entries = 30.6 + 2.8, center time = 85.1 + 10.2 s Data are presented as Mean ± SEM, **p < 0.01.

The locomotor activity of the mice was subsequently tested under mild challenged conditions in the open field during the light phase (Figures 2A,B). Data revealed that female p75KO (compared to WT) mice showed an increased locomotor activity as reflected by increased distance traveled (*t*_(12)_ = −4.0, *p* < 0.01) indicating a novelty seeking behavior. Novelty seeking is associated negatively with risk for depression and anxiety in humans and animal models (Cloninger et al., 1993; Pawlak and Zaremba, 2008; Pawlak et al., 2008). In line with these findings, female p75KO mice displayed higher center distance (*t*_(12)_ = −3.1, *p* < 0.01), center entries (*t*_(12)_ = −3.4, *p* < 0.01) and center time (*p* = 0.06) reflecting a reduction in anxiety. Male p75KO mice were not different from their WT controls.

#### Elevated Plus Maze Test

**Figure 3:**
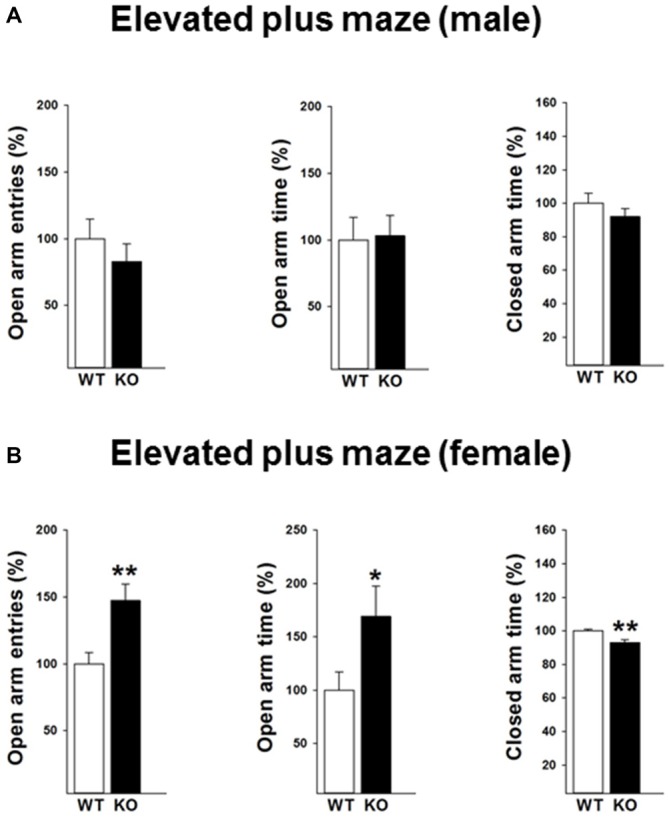
**Reduced anxiety in the elevated plus maze test in the female p75NTR^exon IV−/−^ mice (B).** They show significantly more open arm entries, longer time spent in open arm and less closed arm entries. Results of male mice are depicted in **(A)**. WT: white bar, p75KO: black bar. WT data are normalized to 100%. Group Mean + SEM; Male WT: open arm entries = 9.5 + 1.3, open arm time = 18.4 + 3.1 s closed arm time = 191.9 + 11.3 s, Male p75KO: open arm entries = 7.8 + 1.2, open arm time = 19.3 + 2.8 s closed arm time = 176.4 + 9.4 s, Female WT: open arm entries = 7.1 + 0.6, open arm time = 13.3 + 2.3 s, closed arm time = 254.4 + 2.7.3 s, Female p75KO: open arm entries = 10.5 + 0.8, open arm time = 22.3 + 3.7 s, closed arm time = 236.6 + 4.1 s Data are presented as Mean ± SEM, *p < 0.05; **p < 0.01.

We next performed the elevated plus maze test to validate the findings of the open field test. A reduced anxiety-like behavior in female p75KO mice was further confirmed in the elevated plus maze test as indicated by a significant higher number of open arm entries (*t*_(12)_ = −3.2, *p* < 0.01) an increased time spent in the open arm (*t*_(12)_ = −2.2, *p* < 0.05). Furthermore, the female p75KO mice spent less time in the closed arm further pointing towards a reduction in anxiety (*t*_(12)_ = −3.6, *p* < 0.01). Confirming the open field results there was no difference between male p75KO and WT controls (Figures 3A,B).

#### Flex Field Test

**Figure 4:**
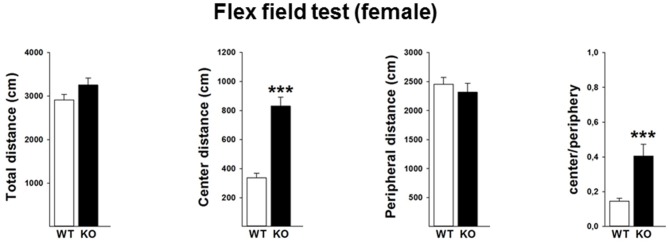
**Reduced level of anxiety without the overall locomotion being affected in the female p75NTR^exon IV−/−^ mice.** WT: white bar, p75KO: black bar. There is no difference in the overall locomotor activity as well as distance traveled in the peripheral part of the arena. Animals show significantly higher center distance as well as higher center to periphery ratio. Data are presented as Mean ± SEM, ***p < 0.001.

In order to validate the locomotor activity in the female mice, we further performed the flex field test in a separate cohort of mice. Data revealed that female p75KO (compared to WT) mice showed no differences in the overall locomotor activity (*t*_(30)_ = −1.7, *p* > 0.05) as well as distance traveled in the peripheral part of the arena (*t*_(30)_ = −0.6, *p* > 0.05). Further analysis indicates that female p75KO displayed a higher center distance (*U* = 5, *p* < 0.001) as well as higher center to periphery ratio (*U* = 13, *p* < 0.001). These data clearly indicate that female p75KO mice display reduced level of anxiety without the overall locomotion being affected (Figure 4).

### Histology

**Figure 5:**
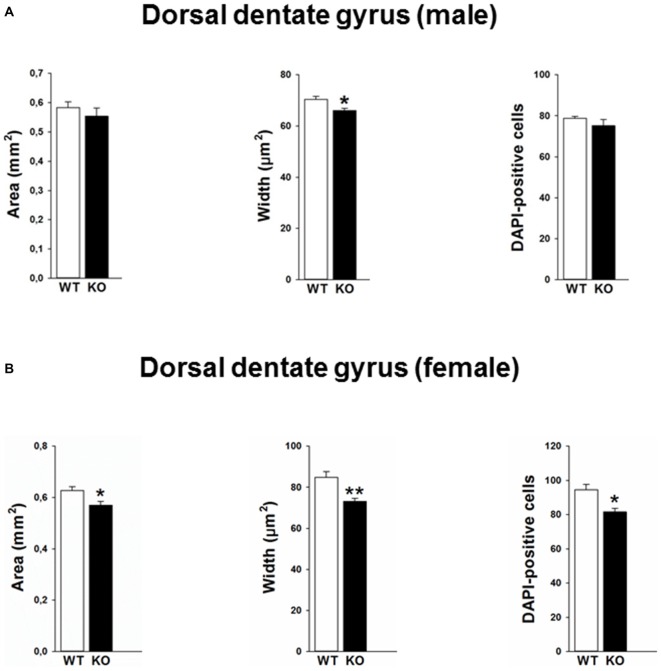
**Altered morphology in the dorsal dentate gyrus of the p75NTR^exon IV−/−^ mice.** Male p75KO mice show a reduced width of the dentate gyrus as compared to WT mice, whereas area of dentate gyrus and number of DAPI labeled cells in the granular layer are comparable **(A).** Female p75KO mice show a significant reduction in all three parameters depicted **(B)**. WT, white bar; p75KO, black bar. Data are presented as Mean ± SEM, *p < 0.05; **p < 0.01.

**Figure 6:**
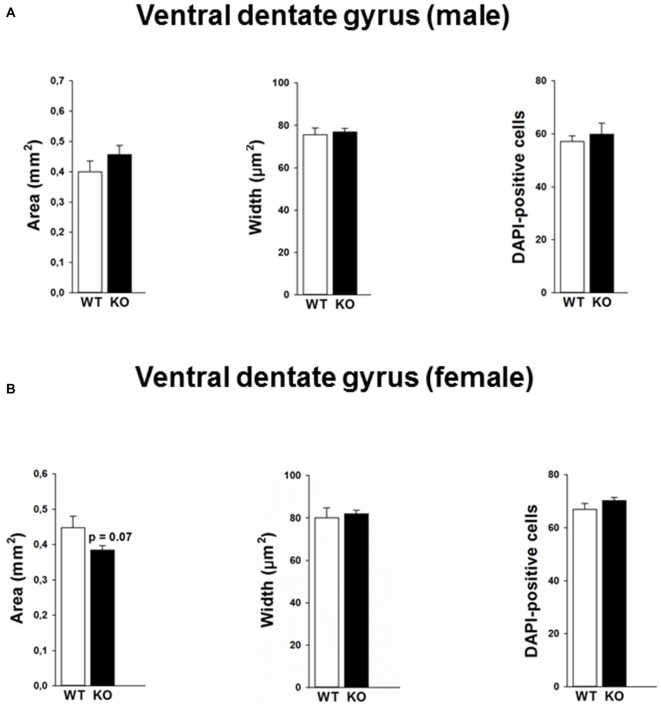
**Morphology in the ventral dentate gyrus of the p75NTR^exon IV−/−^ mice and number of DAPI labeled cells is not altered between p75KO and WT mice for both male (A) and female (B) animals.** However, there is a trend for smaller area of the dentate gyrus in p75KO as compared to WT controls in the female mouse group **(B)**. WT, white bar; p75KO, black bar. Data are presented as Mean ± SEM.

Mutations in the p75NTR have been shown to be associated with changes in dentate gyrus morphology. We confirmed these alterations in our p75KO mice. More specifically, female p75KO mice displayed a reduced area (*p* < 0.05) and smaller width (*p* < 0.01) of the granular cell layer in the dorsal dentate gyrus. In addition, reduced number of DAPI-positive nuclei was also observed in female p75KO mice indicating lower number of cells within the granular layer of the dorsal dentate gyrus (*p* < 0.05). For male p75KO mice alterations were restricted to a smaller width (*p* < 0.05) of the granular cell layer in the dorsal dentate gyrus. Significant results concerning the dorsal dentate gyrus are summarized in Figures 5A,B, for the ventral dentate gyrus in Figures 6A,B. For detailed morphometry results, see “Supplementary Tables 1, 2”.

Furthermore, we performed a correlation analysis between anxiety parameters and morphometric analysis obtained within the dentate gyrus. Our findings reveal that area of the dorsal dentate gyrus was negatively correlated against center distance (*r^2^* = −0.58, *p* < 0.05) and center entries (*r^2^* = −0.54, *p* < 0.05) in the open field test. We also found that the number of DAPI was negatively correlated with the center entries (*r^2^* = −0.54, *p* < 0.05).

## Discussion

The aim of the present study was to characterize general locomotor and anxiety-related behavior and related hippocampal morphometry in male and female p75NTR^exon IV−/−^ mice. The effects of genetic alteration in the p75NTR locus on hippocampal structures and hippocampus-related behavior were looked at in a number of studies with sometimes contradictory results, see Dokter et al. (2015) for discussion.

Most of the studies on behavior of p75NTR-mutants have been performed in a mouse model developed by Lee et al. (1992), where exon III of the p75NTR gene was knocked out (Lee et al., 1992). In this model, the short isoform of the P75NTR arising from alternative splicing of exon III is left intact. von Schack et al. (2001) developed a p75NTR^exon IV−/−^ mouse model lacking both isoforms, leading to severe nervous system defects as well as malformations of blood vessels and an abnormal waddling gait.

In part, the observed discrepancies in behavioral experiments with p75NTR mutants might be explained by the use of different genetic models and background between the animals tested. In addition most of the studies were performed solely in male mice. It is widely recognized that strain as well as sex differences exist in behavioral tests for mood disorders (Palanza, 2001; An et al., 2011). In particular, a lower level of anxiety has been described in the elevated plus maze test for male vs. female c57BL6 mice (An et al., 2011) emphasizing the need to study sex-related differences in behavior of p75NTR mutants.

In our study, general locomotion of male p75NTR^exon IV−/−^ mice did not differ in a familiar homecage environment from wildtype animals. However in female p75NTR^exon IV−/−^ -animals home cage activity was reduced in the active nocturnal phase but not during the light phase. An increased locomotor activity was measured for p75NTR^exon IV−/−^ animals in the open field test, indicative of a novelty seeking behavior. This behavioral trait is positively associated with vulnerability for substance abuse, and negatively with risk for depression and anxiety in humans and animal models (Cloninger et al., 1993; Pawlak and Zaremba, 2008; Pawlak et al., 2008). A reduction of anxiety-like as well as anxiolytic-like behavior for these female animals was also confirmed in an independent elevated plus maze test and with a different set of female p75NTR^exon IV−/−^ mice in a flex field activity system. Therefore three different tests confirm sex specific behavioral differences in p75NTR^exon IV−/−^ mutant animals.

Literature gives contradicting results regarding locomotor activity in adult male p75NTR^exon III−/−^ mice, showing either increased (Peterson et al., 1999) or decreased (Barrett et al., 2010) degrees of activity. Also tests aimed to analyze anxiety-associated behavior in p75NTR^exon III−/−^ mice came to contradictory results, with increased anxiety in a novelty-suppressed feeding test, no differences between genotypes in a forced-swim test, light dark test and elevated plus maze test and anxiolytic effects in the tail suspension test (Catts et al., 2008). Martinowich et al. (2012) reported that there is no difference in baseline depressive-like behavior, such as the forced swim test and tail suspension test but an increase in baseline anxiety-like behavior, such as the elevated plus maze test and open-field test. In our hands, male p75NTR^exon IV−/−^ and control mice exhibit no difference in general locomotor behavior and anxiety-related paradigms, which argues against a confounding effect in our study due to peripheral sensory deficits in the p75NTR^exon IV−/−^ animals.

Fear and anxiety-related behavior is mainly associated with the ventral hippocampus (Kjelstrup et al., 2002; Bannerman et al., 2003a,b; Pentkowski et al., 2006; Calfa et al., 2007; Nascimento Häckl and Carobrez, 2007) as observed after specific ventral lesions, whereas the dorsal hippocampus is likely to be more involved in spatial learning. In a histological study using male p75NTR^exon IV−/−^ mice, Poser et al. (2015) described thickening of the granular and molecular layer of the dentate gyrus, explained by increased cholinergic innervation and imbalance between neurogenesis and apoptosis in the subgranular layer. In the study by Catts et al. (2008) anxiety-like behavior has been analyzed and documented changes in p75NTR^exonIII−/−^ were partially attributed to reduced neurogenesis in the dentate gyrus of knockout animals, which led to reduced dentate gyrus volume. In contrast, Dokter et al. (2015) described an enlargement of the dentate gyrus, mainly due to increase in width of the granular cell layer. This was also seen in p75NTR^exon IV−/−^ mice by Poser et al. (2015). However, the subdivision in ventral and dorsal hippocampus was not taken into an account.

In the present study, we have tested the effect of p75NTR^exon IV−/−^ mutation on the morphology of the male and female hippocampus. Cross sectional areas of dorsal and ventral hippocampal formation were analyzed separately. The area and width of the dorsal dentate gyrus as well as width of the granular cell layer were significantly reduced for female p75NTR^exon IV−/−^ mice, displaying in addition a decreased number of DAPI-stained cells in the granular cell layer. Male p75NTR^exon IV−/−^ mice exhibit a reduction in width of dorsal dentate gyrus. Therefore our study demonstrates that the p75NTR^exon IV−/−^ mutation affects morphology of the hippocampus not only in male but also female mice. The pronounced effect in dorsal hippocampus indicates that spatial memory functions would most likely be affected in female p75NTR^exon IV−/−^ mice.

Anxiolytic behavior has so far been experimentally obtained by lesions of the ventral hippocampus (Kjelstrup et al., 2002; Pentkowski et al., 2006). Therefore our behavioral results in female knockout animals led us to the expectation of more pronounced morphological changes in the ventral part of the hippocampus. However, morphometric analyses revealed no influence of the genetic mutation on ventral dentate gyrus area and width of the granular cell layer. Hence our data that support an influence of p75NTR on anxiety-like behavior in female mice cannot be straightforwardly correlated with changes in ventral hippocampal cross sectional morphology as can be detected by the morphological analyses used in the present study.

In conclusion, our findings show that germ-line deletion of p75NTR has a gender dependent effect on anxiety-like behavior. Alterations in anxiety-related behavior are associated with morphometric alterations in the dorsal, but not ventral, dentate gyrus in the female mice.

## Author Contributions

ZP, AS, NS and GD designed the experiments. ZP, AS and IG performed the experiments. ZP, AS and IG analyzed the data. ZP and AS wrote the article. AS, NS and GD revised the manuscript and approved the final version.

## Funding

This work was supported by research grants from the Austrian Science Fund (FWF): ZFW012060, W1206-B18 and B-16 SPIN and Sonderforschungsbereich F4410 (University of Innsbruck). AS was supported by the Graduate Program “Signal Processing in Neurons” (SPIN).

## Conflict of Interest Statement

The authors declare that the research was conducted in the absence of any commercial or financial relationships that could be construed as a potential conflict of interest.
