# Supplementary figures and images for: Reduced Anxiety-Like Behavior and Altered Hippocampal Morphology in Female p75NTRexon IV−/− Mice
Source: Front Behav Neurosci. 2016 Jun 1;10:103. doi: 10.3389/fnbeh.2016.00103 (PMC4887477; doi:10.3389/fnbeh.2016.00103)

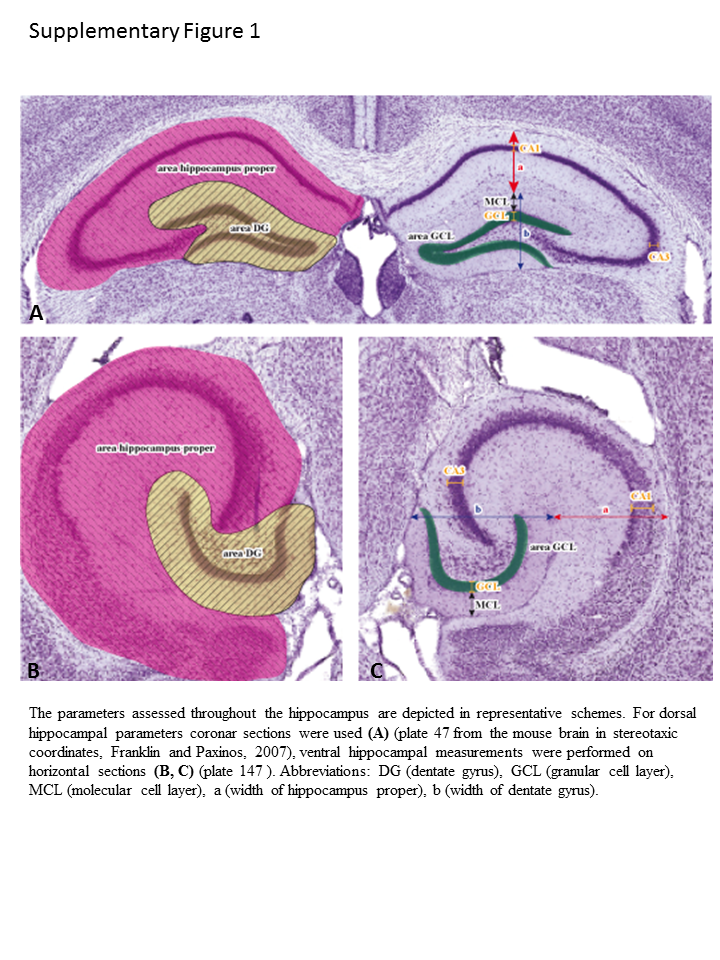

Supplement: Supplementary file 1 [file Image_1.tif]
