# Supplementary material for: Reduced Anxiety-Like Behavior and Altered Hippocampal Morphology in Female p75NTRexon IV−/− Mice
Source: Front Behav Neurosci. 2016 Jun 1;10:103. doi: 10.3389/fnbeh.2016.00103 (PMC4887477; doi:10.3389/fnbeh.2016.00103)
Supplement: Supplementary file 2 [file Table_1.docx]

Supplementary Table 1

Morphometry dorsal hippocampus

| DORSAL | area HC-DG  (mm^2^) | CA1  (µm) | CA3  (µm) | area DG  (mm^2^) | area GCL  (mm^2^) | width GCL  (µm) | width  MCL  (µm) | a  (µm) | b  (µm) | a/b |
| --- | --- | --- | --- | --- | --- | --- | --- | --- | --- | --- |
| male-wt | 1.61±  0.05 | 49.49± 1.32 | 128.01± 4.95 | 0.58±  0.02 | 0.12± 0.002 | 70.36 ±  1.23 | 187,68±  1,38 | 544.87± 7.21 | 552.38± 9.28 | 1.03  ± 0.03 |
| male-ko | 1.48±  0.04 | 48.25± 1.67 | 115.53± 3.21 | 0.55±  0.03 | 0.11± 0.002 | 66.11 ±  0.80 | 188,69±  2,52 | 509.54± 6.59 | 521.08± 17.40 | 0.99  ± 0.05 |
| female- wt | 1.65±  0.08 | 56.74± 1.72 | 134.65± 10.01 | 0.63± 0.01 | 0.14± 0.005 | 84.86± 2.73 | 187,78±  2,91 | 544.70± 12.50 | 603.88± 7.71 | 0.93± 0.02 |
| female- ko | 1.54±  0.05 | 54.81± 1.58 | 126.56± 6.26 | 0.57± 0.01 | 0.13± 0.004 | 73.15± 1.47 | 181,05±  4,07 | 532.74± 10.34 | 522.14± 11.64 | 1.05± 0.03 |

Supplementary Table 2

Morphometry ventral hippocampus

| VENTRAL | Area  HC-DG  (mm^2^) | CA1  (µm) | CA3  (µm) | area DG  (mm^2^) | area GCL  (mm^2^) | width GCL  (µm) | width  MCL  (µm) | a  (µm) | b  (µm) | a/b |
| --- | --- | --- | --- | --- | --- | --- | --- | --- | --- | --- |
| male-wt | 2.18±  0.03 | 362.12±  29.19 | 97.59± 3.29 | 0.40± 0,03 | 0.06± 0.004 | 75.84± 3.37 | 209.71±  7.47 | 909.95± 9.29 | 763.12± 42.24 | 1.31± 0.08 |
| male-ko | 2.22±  0.04 | 219.70±  17.86 | 96.07± 1.23 | 0.46± 0.03 | 0.07± 0.004 | 76.86± 1.78 | 191.00±  2.73 | 856.19± 4.84 | 811.43± 25.86 | 1.14± 0.07 |
| female-wt | 2.34±  0.06 | 249.25±  15.74 | 102.95± 2.25 | 0.45± 0.03 | 0.07± 0.005 | 80.1±  4.74 | 187.15±  5.14 | 965.52± 4.91 | 828.9± 26.18 | 1.28±  0.1 |
| female-ko | 2.12±  0.03 | 238.44±  5.89 | 100.1± 1.54 | 0.38± 0.01 | 0.06± 0.001 | 82.07±  1.6 | 178.97±  3.86 | 902.21± 8.9 | 754.39± 15.29 | 1.3±  0.03 |
